# Mesenchymal stem cells generate a CD4^+^CD25^+^Foxp3^+^ regulatory T cell population during the differentiation process of Th1 and Th17 cells

**DOI:** 10.1186/scrt216

**Published:** 2013-06-04

**Authors:** Patricia Luz-Crawford, Monica Kurte, Javiera Bravo-Alegría, Rafael Contreras, Estefania Nova-Lamperti, Gautier Tejedor, Danièle Noël, Christian Jorgensen, Fernando Figueroa, Farida Djouad, Flavio Carrión

**Affiliations:** 1 Inserm, U 844, Montpellier F-34091, France; 2 Université MONTPELLIER1, UFR de Médecine, Montpellier F-34000, France; 3 Service d'Immuno-Rhumatologie, Hôpital Lapeyronie, Montpellier F-34295, France; 4 Laboratorio de inmunologia celular y molecular, Universidad de los Andes, San carlos de apoquindo 2200, CP. 7620001, Las Condes, Santiago, Chile; 5 Programa Doctorado en Biotecnología, Universidad Santiago de Chile. Avenida Libertador Bernardo O’Higgins 3363, CP. 9170022, Estación Central, Santiago, Chile

**Keywords:** Mesenchymal stem cells, Th1, Th17, Immunosuppression, CD4^+^CD25^+^Foxp3^+^ T cells

## Abstract

**Introduction:**

Mesenchymal stem cells (MSCs) are adult, multipotent, stem cells with immunomodulatory properties. The mechanisms involved in the capacity of MSCs to inhibit the proliferation of proinflammatory T lymphocytes, which appear responsible for causing autoimmune disease, have yet to be fully elucidated. One of the underlying mechanisms studied recently is the ability of MSCs to generate T regulatory (Treg) cells *in vitro* and *in vivo* from activated peripheral blood mononuclear cells (PBMC), T-CD4+ and also T-CD8+ cells. In the present work we investigated the capacity of MSCs to generate Treg cells using T-CD4+ cells induced to differentiate toward the proinflammatory Th1 and Th17 lineages.

**Methods:**

MSCs were obtained from mouse bone marrow and characterized according to their surface antigen expression and their multilineage differentiation potential. CD4^+^ T cells isolated from mouse spleens were induced to differentiate into Th1 or Th17 cells and co-cultured with MSCs added at day 0, 2 or 4 of the differentiation processes. After six days, CD25, Foxp3, IL-17 and IFN-γ expression was assessed by flow cytometry and helios and neuropilin 1 mRNA levels were assessed by RT-qPCR. For the functional assays, the ‘conditioned’ subpopulation generated in the presence of MSCs was cultured with concanavalin A-activated CD4+ T cells labeled with carboxyfluorescein succinimidyl ester. Finally, we used the encephalomyelitis autoimmune diseases (EAE) mouse model, in which mice were injected with MSCs at day 18 and 30 after immunization. At day 50, the mice were euthanized and draining lymph nodes were extracted for Th1, Th17 and Treg detection by flow cytometry.

**Results:**

MSCs were able to suppress the proliferation, activation and differentiation of CD4^+^ T cells induced to differentiate into Th1 and Th17 cells. This substantial suppressive effect was associated with an increase of the percentage of functional induced CD4^+^CD25^+^Foxp3^+^ regulatory T cells and IL-10 secretion. However, using mature Th1 or Th17 cells our results demonstrated that while MSCs suppress the proliferation and phenotype of mature Th1 and Th17 cells they did not generate Treg cells. Finally, we showed that the beneficial effect observed following MSC injection in an EAE mouse model was associated with the suppression of Th17 cells and an increase in the percentage of CD4^+^CD25^+^Foxp3^+^ T lymphocytes when administrated at early stages of the disease.

**Conclusions:**

This study demonstrated that MSCs contribute to the generation of an immunosuppressive environment via the inhibition of proinflammatory T cells and the induction of T cells with a regulatory phenotype. Together, these results might have important clinical implications for inflammatory and autoimmune diseases.

## Introduction

Mesenchymal stem cells (MSCs) are multipotent stromal cells characterized by their ability to differentiate into cells from mesodermal tissue making them an interesting cell source for application in regenerative medicine [1,2]. Another attractive potential of MSCs is their capacity to inhibit the proliferation of T and B lymphocytes, natural killer and dendritic cells both *in vitro* and *in vivo*[3-6]*.* These immunosuppressive abilities are mediated by different mechanisms specific for human or mouse MSCs, such as indoleamine 2,3-dioxygenase (IDO) or nitric oxide (NO), respectively, or overlapping suppressive factors, such as transforming growth factor β1 (TGF-β1), prostaglandin E2 (PGE2) and IL-10 among others [7-9]. Moreover, it has been shown that MSCs are able to generate CD4^+^CD25^+high^ Foxp3^+^ T regulatory (Treg) cells *in vitro,* from activated human peripheral blood mononuclear cells (PBMC), mouse splenocytes or isolated T-CD4 cells. Indeed, MSCs promote the induction of CD4^+^CD25^+high^ regulatory cells from human PBMC cells activated with IL-2 [10]. In the same line, Maccario *et al*. demonstrated that MSCs favor the differentiation of CD4^+^ T-cell subsets co-expressing CD25 and/or CTLA4, two markers of Treg cells [11]. These observations were supported and extended by a study showing that direct MSC-T cell contact is required for Foxp3 and CD25^High^ expression by CD4^+^ T cells; however, soluble factors produced by MSCs, such as TGF-β1 and PGE2, also played a non-redundant contribution in the generation of CD4^+^CD25^+^Foxp3^+^[8]. A role for other molecules including the human leukocyte antigen-G5 (HLA-G5) and the stress inducible enzyme heme-oxygenase-1 (HO-1) has also been described in the generation of regulatory T cell phenotype mediated by human MSCs [12,13]. The ability of MSCs to induce such a regulatory phenotype in T cells was described both *in vitro* and *in vivo*. As an example, in a mouse animal model of inflammatory bowel disease (IBD), the beneficial effect of injected human adipose derived MSCs (hASCs) on the clinical and histological scores of mice was associated with an increased number of CD4^+^CD25^+^Foxp3^+^ and CD4^+^IL10^+^ cells in the lymph nodes [14]. The induction of a regulatory T cell phenotype population has also been shown in clinical applications. Indeed, a significant induction of CD4^+^CD25^+^Foxp3^+^ cells was observed in two systemic lupus erythematous (SLE) patients [15]. Moreover, after autologous MSC administration in patients with kidney transplantation, Perico *et al*. reported not only an increase in the number of CD4^+^CD25^+^Foxp3^+^ cells but also a substantial decrease of the activity of CD4^+^ and CD8^+^ effectors as well as a subsequent improvement in their renal function [16].

In the context of inflammatory diseases, T helper 1 (Th1) and Th17 T cell subsets are well known to mediate inflammation [17,18]. Interestingly, we showed that MSCs inhibit human Th17 cell differentiation and function and induce a regulatory T cell phenotype [19]. This result revealed that, even under inflammatory conditions, MSCs exert *in vitro* anti-inflammatory effects through the induction of a regulatory T cell phenotype. However, the capacity of MSCs to generate functional Treg cells *in vitro* during the differentiation process or on fully differentiated Th1 and Th17 cells still remains to be elucidated. Therefore, in this study, we explored the capacity of MSCs to generate, *in vitro,* functional CD4^+^CD25^+^Foxp3^+^ Treg cells under Th1 and Th17 inflammatory culture conditions. In parallel, in the experimental autoimmune encephalomyelitis (EAE) model, we assessed the percentage of regulatory T cells after MSC administration at two different time points post-immunization. The aim of this study was to determine whether MSCs are able to increase the percentage of regulatory T cells *in vitro* when co-cultured with either CD4^+^ cells induced to differentiate into Th1 and Th1 or with fully differentiated Th1 and Th17 cells and *in vivo* in the EAE model.

## Methods

### Isolation and characterization of mouse mesenchymal stem cells

MSCs were isolated from eight- to ten-week-old C57BL/6 mice. Bone marrow cells were collected by flushing femurs and tibias and the cell suspension (1 × 10^6^cells/cm^2^) was plated in a modified minimum essential Eagle's medium (MEM) © (α-MEM, Gibco, Auckland, NZ) supplemented with 20% fetal bovine serum (FBS) (Hyclone, Thermo Fisher Scientific, Brebières, France), 2 mM glutamine and 100 U/mL penicillin with 100 mg/mL streptomycin (Gibco, Auckland, NZ) (α-20). At sub-confluence, cells were replated at a density of 20,000 cells/cm^2^ and, after the second passage, MSCs were isolated by negative selection using a CD45^+^ microbeads kit (Miltenyi Biotec, Bergisch-Gladbach, Germany). MSCs were characterized for expression of hematopoietic and mesenchymal cell antigens by fluorescence-activated cell sorting (FACS) analysis and by their capacity to differentiate into adipogenic, chondrogenic and osteogenic lineages as previously described [20].

### Th1 and Th17 differentiation and MSC cocultures

CD4^+^ T cells from spleen of C57BL/6 mice were purified by negative selection using the CD4^+^ T cell Isolation Kit MicroBeads (Miltenyi Biotec) according to the manufacturer’s instructions. Purified CD4^+^ T cells were cultured in complete medium containing RPMI 1640 supplemented with 10% heat-inactivated FBS, 2 mM l-glutamine, 100 U/mL penicillin/100 μg/mL streptomycin. In a 24-well plate, 2 × 10^6^ CD4^+^ T cells were cultured in the presence of 2.5 μg/ml coated antibodies against CD3 and 1.5 μg/ml CD28 (BD Biosciences, San Jose, CA, USA) under Th1 or Th17 differentiation conditions. Th1 cells were differentiated with 20 ng/ml of IL-12 (R&D Systems, Minneapolis, MN, USA) and 2.5 μg/ml anti-IL-4 antibodies (BD Biosciences). Th17 cells were differentiated with 50 ng/ml IL-6 (R&D Systems, Minneapolis, MN, USA), 5 ng/ml of TGF-β1 (BioVision, Milpitas, CA USA) and 2.5 μg/ml of anti-IFN-γ and -IL-4 antibodies (BD Biosciences). MSCs were added at a MSC:Th ratio of 1:10 or 1:100 at day 0, 2 or 4 of the Th1 and Th17 differentiation process. After six days of culture, relative cell quantification was measured using the CellTiter-Glo™ luminescent cell viability assay (Promega, Charbonnières-les-Bains, France) and intracellular cytokine detection was measured by flow cytometry.

### Analysis by flow cytometry

After six days of culture, T cells were stimulated for four hours with 50 ng/ml phorbolmyristate acetate (PMA) (Sigma-Aldrich, St Louis, MO, USA) and 1μg/ml ionomycin (Sigma-Aldrich), prior to the addition of 10 μg/ml brefeldin A (eBiosciences, San Diego, CA, USA). For the detection of surface markers, cells were stained with CD4-PEcy5 (eBiosciences) and CD25-PE-texas red (Invitrogen, Grand Island, NY, USA) and incubated for 20 minutes at 4°C in the dark. After two washing steps, we performed intracellular staining for IFN-γ-FITC, IL-17-PE or FoxP3-Alexa488 detection. For that purpose, cells were fixed and permeabilized using the Cytofix/Cytoperm™ (BD Biosciences) kit according to the manufacturer’s instructions. Acquisition was performed with a Coulter Epics-XL flow cytometer using the System II software (Coulter Corporation, Brea, CA, USA). Analysis was performed using the FCS express software (De novo softwares, Los Angeles, CA, USA).

### Functional assay

After six days of co-culture at different times post- T-CD4^+^ activation (0, 2 and 4), the ‘conditioned’ Treg subpopulations generated from Th1 and Th17 cells cocultured with MSCs (cond-Th1 or cond-Th17 cells) were evaluated for their ability to suppress allogenic T cell proliferation. For that purpose, fresh T-CD4^+^ cells were labeled with carboxyfluorescein succinimidyl ester (CFSE) and cocultured with or without cond-Th1 or cond-Th17 cells at different ratios in the presence of 1 μg/ml of concanavalin A (ConA) (Sigma-Aldrich). After 72 hours, the proliferation of CD4^+^ T cells was analyzed on a FACS Canto II using the BD FACSDiva software measured by flow cytometry.

### Quantification of cytokines

Enzyme-linked immunosorbent assay (ELISA) from R&D Systems (Minneapolis, MN, USA) for IL-10 and the Enzyme Immunoassay kit from Arbor Assays (Ann Arbor, MI, USA) for PGE2 were used. IL-10 and PGE2 were quantified from the supernatants of cocultures which were stored at −20°C until tested.

### MSC purification from Th1 and Th17 cocultures

MSCs were washed three times with a PBS/0.05mM ethylenediamine tetraacetic acid (EDTA) buffer in order to detached lymphocytes from MSCs. Then, MSCs were trypsinized and resuspended in α-20 and cultured for two hours. After this time, MSCs were washed three times with a PBS/0.05mM EDTA to eliminate possible resting contamination with lymphocytes. The percentage of MSCs was 98%.

### RT-qPCR analysis

Total RNA from purified MSCs and from Th1 and Th17 cells was extracted in the presence or absence of MSCs using the RNeasy mini kit (Qiagen S.A., Courtaboeuf, France). RNA (500 ng) was reverse transcribed using the Multiscribe reverse transcriptase (Applied Biosystems, Courtaboeuf, France). Quantitative PCR was performed using the SYBR Green I Master kit and a LightCycler® 480 Detection System, following the manufacturer’s recommendations (Roche Applied Science, Meylan, France). Specific primers for IL-10, TGF-β1, Helios, Foxp3 and Nrp1 were designed using the Primer3 software [21]. Briefly, 50 ng cRNA were amplified and the analysis of mRNA expression level was performed using the Roche LightCycler® 480 software 1.5. Expression levels of transcripts were normalized to the housekeeping gene ribosomal protein S9 (RPS9). For quantification, values were expressed as the relative mRNA level of specific gene expression as obtained using the 2^−ΔCt^ method.

### EAE induction and treatment protocols

Female C57BL/6J mice, six- to eight-weeks old, were purchased from the Faculty of Medicine, Universidad de Chile (Santiago, Chile). All animals were housed and treated according to the guidelines of the Animal Ethical Committee of our Institution (Universidad de los Andes, Chile). Mice were immunized according to a previously published protocol using 50 μg MOG35-55 (LifeTein, Detroit, MI, USA), emulsified in Complete Freund’s Adjuvant (CFA; Difco, Detroit, MI, USA) containing 4 mg/ml *Mycobacterium tuberculosis* H35RA (strain H35Ra; Difco, Detroit, MI, USA) and injected subcutaneously [22]. Immunization with MOG35-55 was followed by intraperitoneal administration of 350 ng pertussis toxoid (Sigma-Aldrich) on days 0 and 2. Mice were injected with 1 × 10^6^ MSCs on days 18 and 30 after immunization. Clinical score was recorded daily and assigned according to a standard and validated 0 to 5 scale [23]. At day 50, the mice were euthanized and splenocytes and draining lymph nodes (drLN) were extracted and cultured in RPMI 1640 supplemented with 10% of heat-inactivated FCS, 2 mM L-glutamine and 100 U/mL penicillin/100 μg/mL streptomycin in 24 well plates for four hours with 50 ng/ml PMA and 1 μg/ml ionomycin (Sigma-Aldrich), in the presence of 10 μg/ml brefeldin A (eBiosciences) for intracellular staining.

### Statistical analysis

Results are expressed as the mean ± (SEM). Individual experiments were carried out between three and seven times to ensure reproducibility. Generated *P* values and post-analyses were performed with the Kruskal–Wallis test, considering non-normal distributions with small sample sizes and multiple groups and the Mann–Whitney test to compare between two groups. *P*-values <0.05 (*), P <0.01 (**) or P <0.001 (***) were considered statistically significant. Analysis and graphical representation were performed using Graph-Pad Prism™ software (Graphpad, San Diego, CA, USA).

## Results

### Phenotypic and functional characterization of murine MSCs

**Figure 1:**
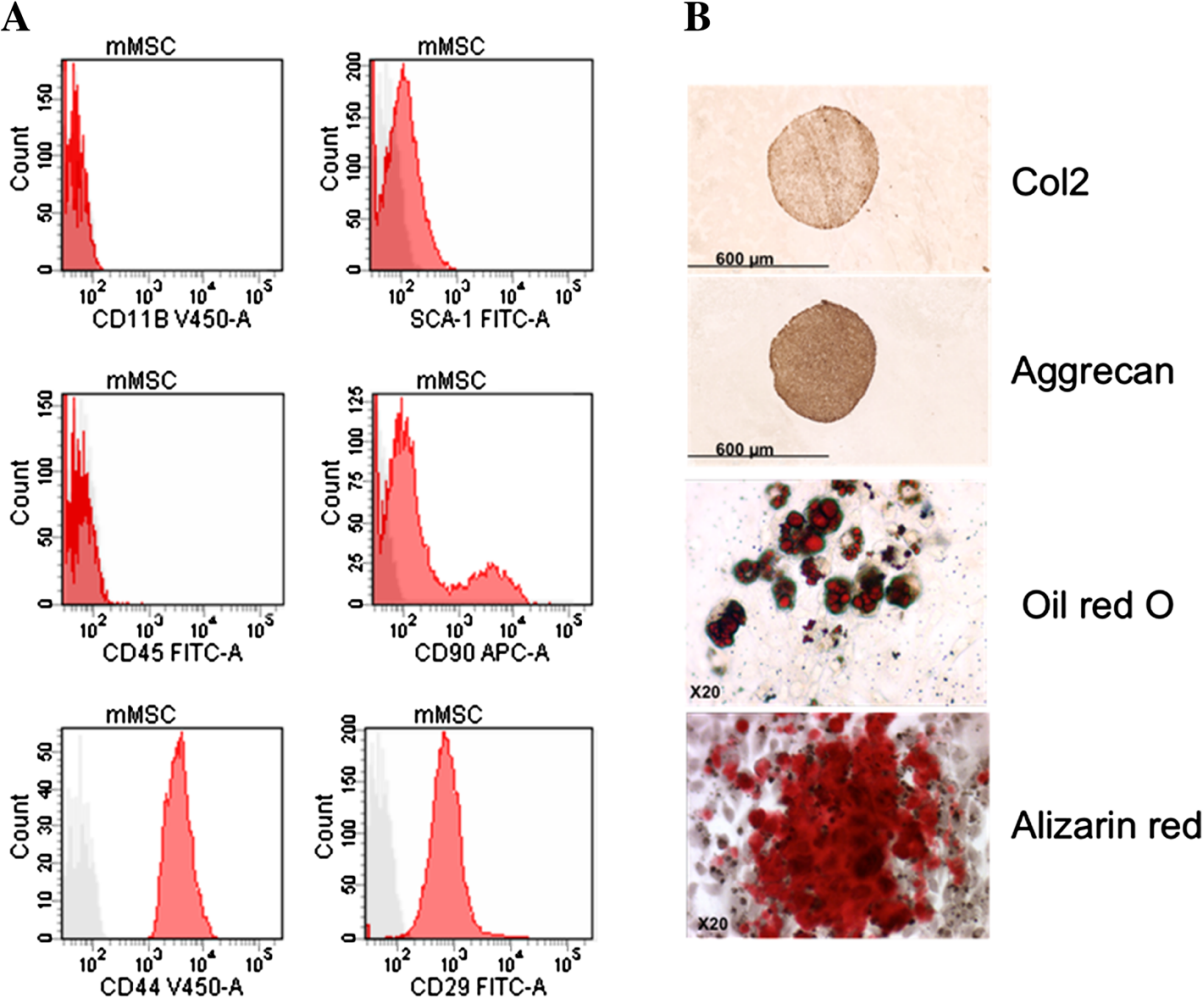
**Surface antigens and differentiation potential of murine mesenchymal stem cells (MSCs). A)** Phenotypic characterization of murine MSCs at passage nine to ten by FACS analysis. A representative histogram for each antigen is shown in red. **B)** Multilineage differentiation potential was assessed by their capacity to differentiate into chondrocyte (Col2 and aggrecan expression, up), adipocyte (Oil Red O staining; middle), and osteoblast (alizarin red S staining; bottom). FACS, fluorescence-activated cell sorting.

Murine bone marrow-derived MSCs were successfully isolated by negative selection with the CD45^+^ microbeads isolation kit. After passage seven, MSCs had a stable fibroblast-like phenotype and were positive for the mesodermal surface antigens CD90, CD29, SCA-1 and CD44 and negative for the hematopoietic markers CD45 and CD11b as shown in Figure 1A. MSCs were also functionally capable of differentiating into adipocytes, chondrocytes and osteoblasts under inductive culture conditions showing their multipotent characteristics (Figure 1B).

### MSCs inhibit the activation, proliferation and differentiation of Th1 and Th17 cells

**Figure 2:**
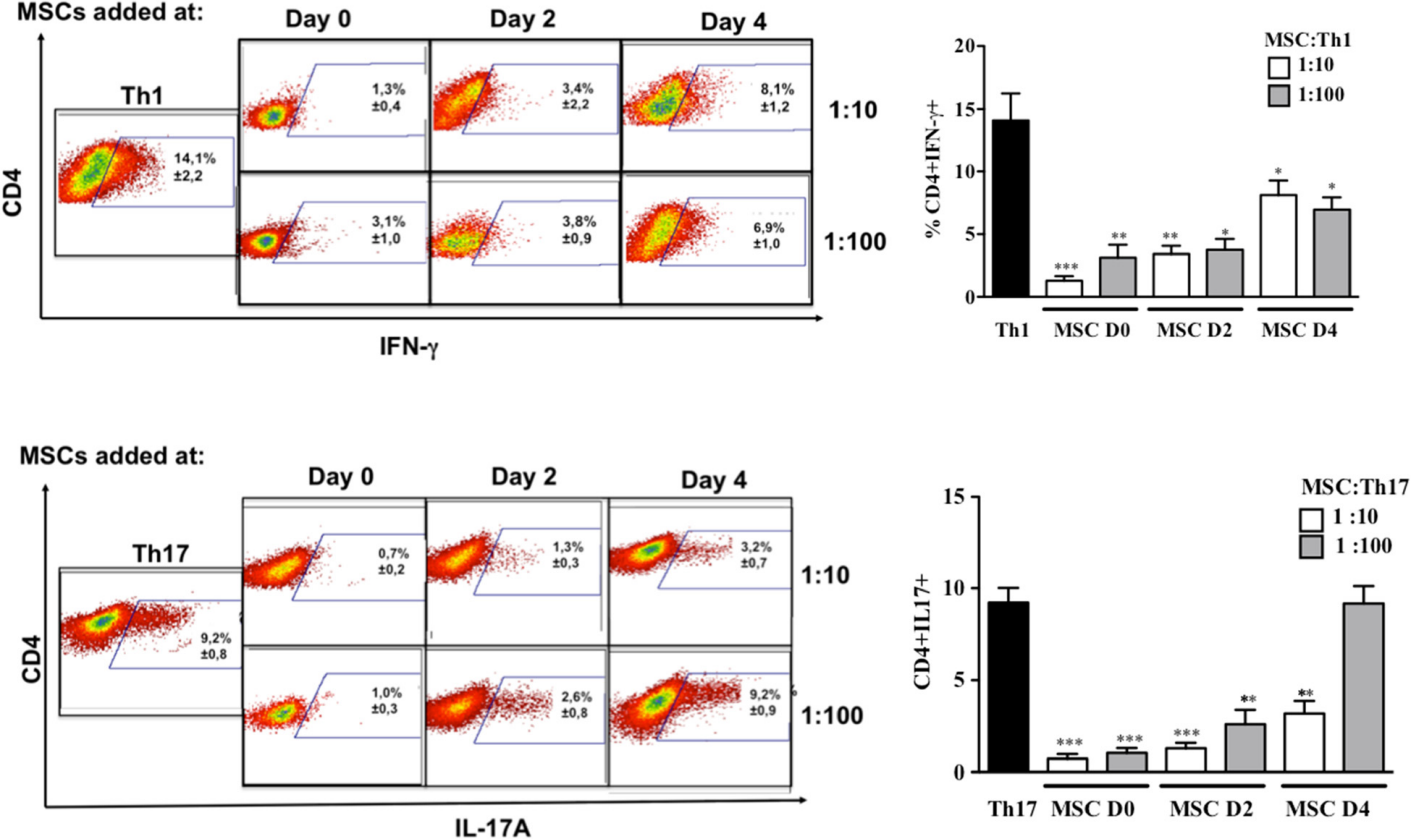
**MSCs inhibit Th1 and Th17 differentiation depending on the stage of activation and cell ratio.** The differentiation of Th1 and Th17 cells was measured by intracellular detection of IFN-γ and IL-17 positive cells, respectively, from purified CD4-T cells differentiated into Th1 and Th17 cells in the presence or absence of MSCs at different stages of activation and different MSC:Th ratios (left: representative dot plot: 1:10 upper panels and 1:100 lower panels). ** = *P* <0.01 and * = *P* <0.05, n = six for Th1 and n = seven for Th17. Values represent means ± SED for six and seven independent experiments for Th1 and Th17 cells, respectively. MSCD0, D2 and D4 = MSC added at day 0, 2 and 4 of the differentiation process, respectively. MSCs, mesenchymal stem cells; Th, T helper.

**Figure 3:**
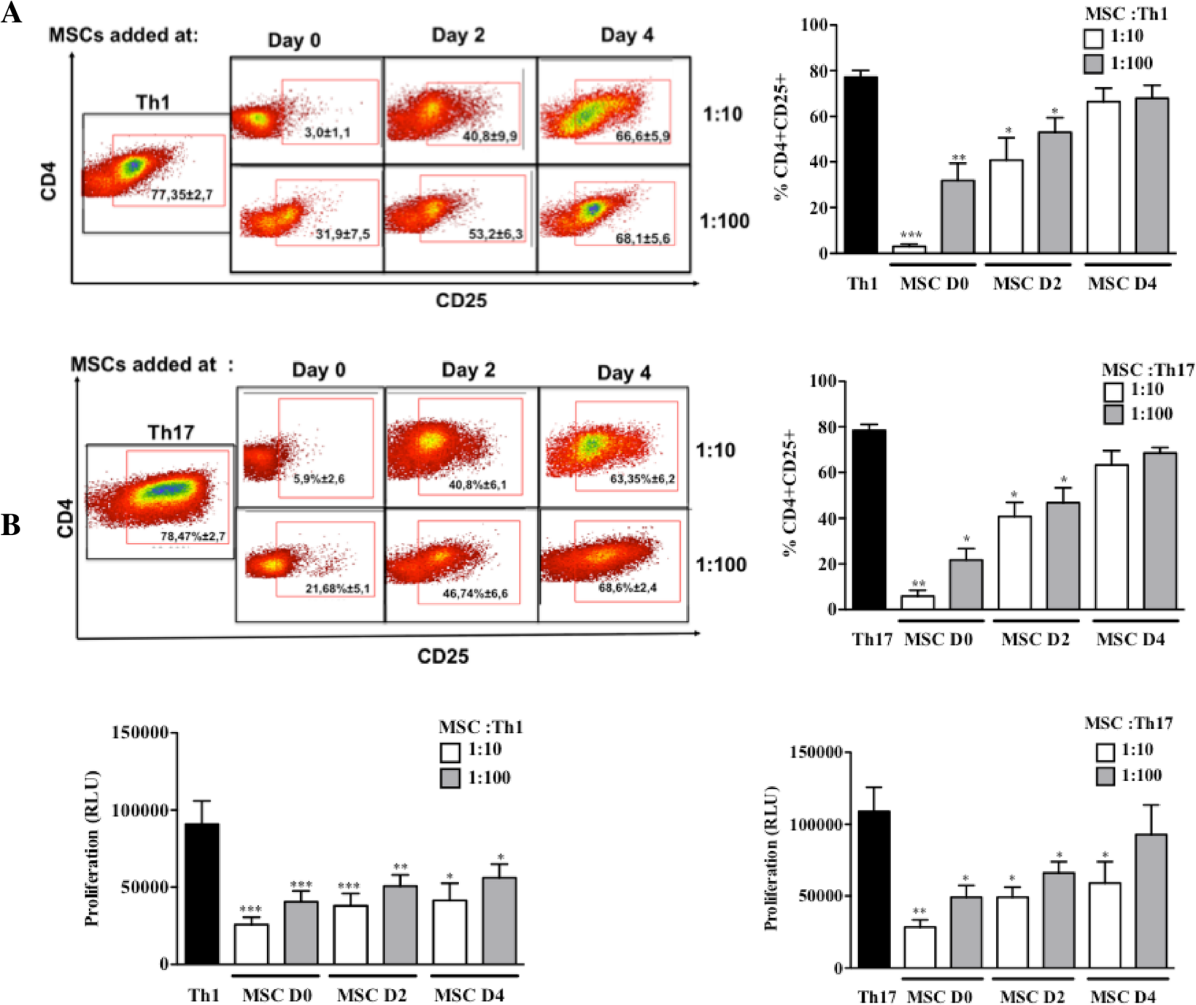
**MSCs inhibit the activation and the proliferation of Th1 and Th17 cells.A)** MSCs inhibit the expression of CD25 in the early stage of activation of Th1 and Th17 cells. T cell activation was measured using purified CD4^+^ T cells differentiated into Th1 or Th17 cells in the presence or absence of MSCs according to the expression of the activation surface antigen CD25 (left: representative dot plot: 1:10 upper panels and 1:100 lower panels). **B)** MSCs suppress Th1 and Th17 cells independent of the stage of activation and cell ratio. Relative cell quantification was determined using purified CD4^+^ T cells differentiated into Th1 or Th17 cells in the presence or absence of MSCs added at day 0, 2 or 4 after T cell activation and at a MSCs:Th ratio (1:10 and 1:100), using the Cell Titer Glo™ luminescence assay. RLU = relative light units**.** MSCs, mesenchymal stem cells; Th, T helper.

The effect of MSCs on the activation, proliferation and Th1 or Th17 differentiation was assessed using purified CD4^+^ T cells activated with anti-CD3/CD28 monoclonal antibodies (mAb) under Th1 or Th17 skewing conditions. MSCs were added at day 0, 2 or 4 post-activation. First, we showed that under Th1 and Th17 culture conditions, IFN-γ-producing cells and IL-17-producing cells, respectively, were generated after six days of culture (Figure 2). Under these conditions, the addition of MSCs at the beginning of the differentiation process (day 0) highly reduced the number of activated CD4^+^CD25^+^ T cells, whatever the ratio used (Figure 3A). This effect was still significant when MSCs were added at day 2. Interestingly, while MSC addition did not affect the activation state of fully differentiated Th1 and Th17 cells, they significantly suppressed the latter mature T cells when added at the highest ratio (Figure 3B). We then studied the effect of MSCs on the T cell differentiation process toward Th1 and Th17 and observed a significant inhibition of the percentage of IFN-γ-producing Th1 and IL-17-producing Th17 cells. However, the percentage of IL-17-producing mature Th17 cells co-cultured with MSCs was not affected at the lowest MSC:T-cell ratio tested (Figure 2).

### MSCs induce functional CD4^+^CD25^+^Foxp3^+^ T cells during the differentiation process of Th1 and Th17 cells

**Figure 4:**
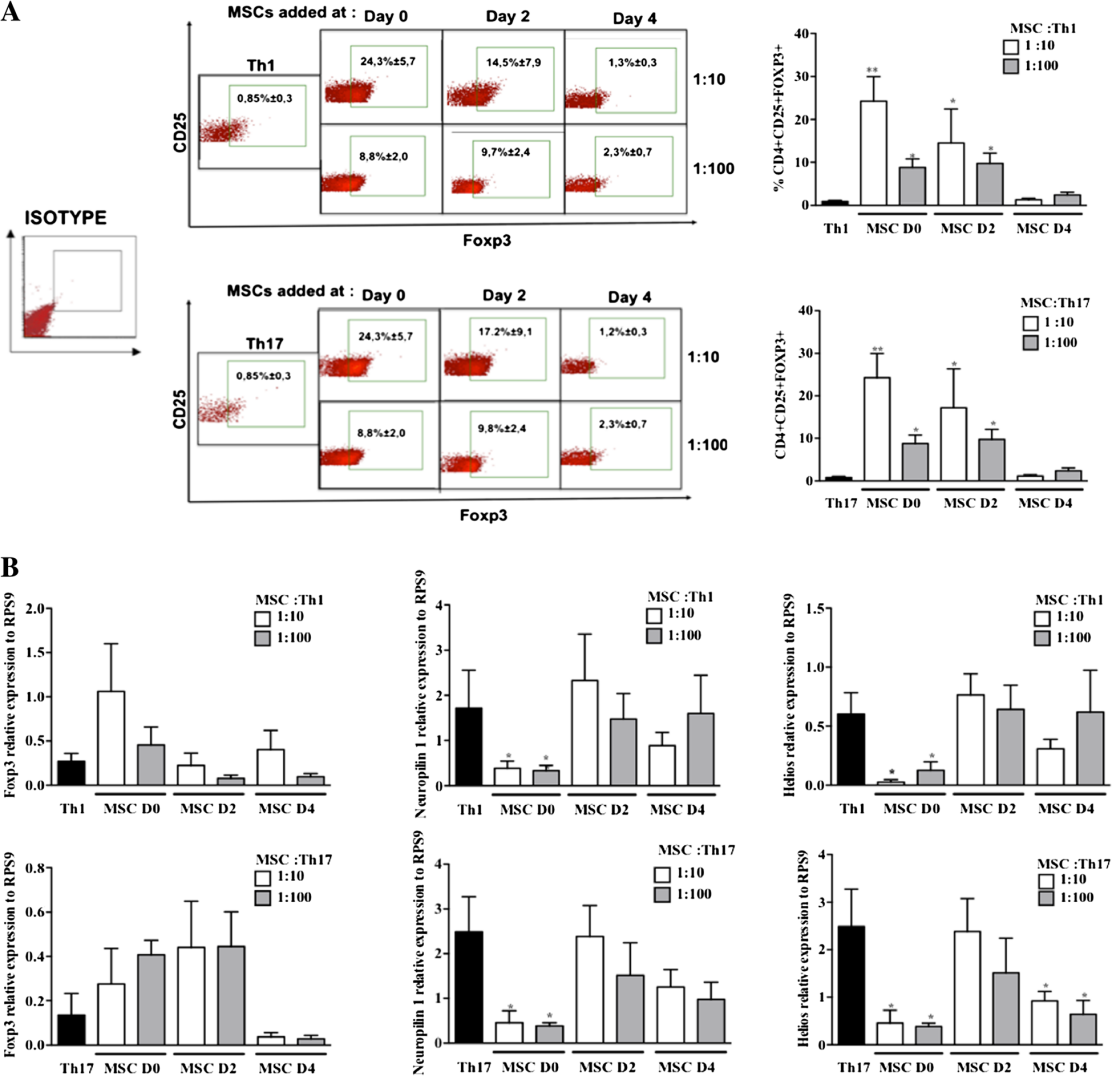
**MSCs generated iTreg cells during the differentiation process of Th1 and Th17 cells. A)** Treg generation from Th1 and Th17 cells cocultured with MSCs at different stages of activation and MSC:Th ratios. Treg phenotype was determined according to the percentage of CD4 + CD25+ cells that express Foxp3 by flow cytometry (Dot plot upper A for Th1 and Upper B for Th17). **B)** Foxp3, Nrp 1 and helios mRNA expression levels weres quantified from Th1 and Th17 cells cocultured in the presence or absence of MSCs at different stage of activation and MSC:Th ratios (** = *P* <0.01 and * = *P* <0.05 ). iTreg, induced Treg; MSCs, mesenchymal stem cells; Nrp 1, neuropilin 1; Th, T helper; Treg, regulatory T cells.

**Figure 5:**
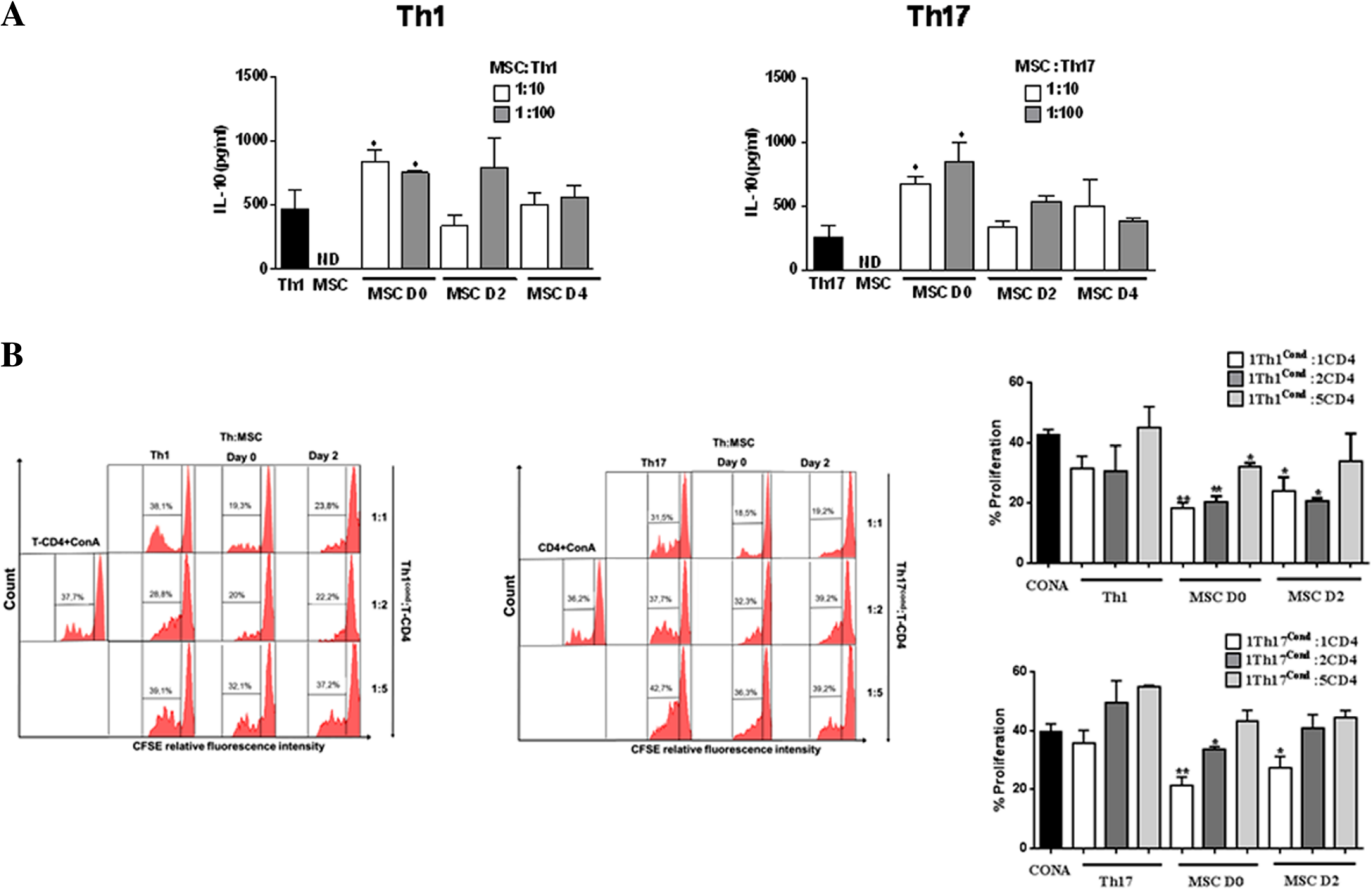
**IL-10 production from supernatants of coculture of Th1 and Th17 cells with MSCs and functional assay.(A)** IL-10 production was quantified in the supernatants of Th1 and Th17 cells co-cultured in the presence or absence of MSCs. * = *P* <0.05, comparing to Th1 and Th17 cells as control group. All the values represent means ± SED of three independent experiments. **B)** Functional effect of iTreg cells generated using Th1 and Th17 cells in cocultures with MSCs (conditioned Th1 or Th17, Th1^cond^ or Th17^cond^, respectively). The suppressive capacity of Th1^cond^ or Th17^cond^ was assessed by following the number of cell divisions of ConA-stimulated T cells, which were labeled by CFSE. Different ratios of Th^cond^:CD4^+^ (1:1; 1:2 or 1:5) were tested. ** = *P* <0.01 and * = *P* <0.05. All the values represent means ± SED of three independent experiments for Th1 and Th17 cells. Th1^cond^ and Th17 ^cond^ = Th1 and Th17 cells that were co-cultured with MSCs added at different time points of the differentiation processes. CFSE, carboxyfluorescein succinimidyl ester; ConA, concanavalin A; iTreg, induced regulatory T cells; MSCs, mesenchymal stem cells; Th, T helper.

Previous studies have shown that MSCs induce CD4^+^CD25^+^Foxp3^+^ T regulatory cell phenotype from PBMC or CD4^+^ activated cells *in vitro*[8,10,11]. In the present study, we investigated the capacity of MSCs to induce functional Treg cells when co-cultured with T cells induced to differentiate toward Th1 or Th17 cells. Flow cytometry analysis revealed that MSCs increased the percentage of CD4^+^CD25^+^Foxp3^+^ T cells only when they were added at day 0 or 2 of the differentiation processes (Figure 4A). This latter increase of Foxp3 was confirmed at the RNA level (Figure 4B). Moreover, we showed an up-regulation of IL-10 production in the supernatants (Figure 5A). Since we demonstrated that MSCs suppressed the proliferation of CD4^+^T cells undergoing Th1 or Th17 differentiation we hypothesized that MSCs generate induced CD4^+^CD25^+^Foxp3^+^ T cells. To test that hypothesis, we investigated whether the CD4^+^CD25^+^Foxp3^+^ T cells obtained in the co-cultures with MSCs were natural (nTregs) or induced (iTregs) by determining the expression levels of surface Nrp1 and helios, an ikaros family transcription factor [24-26]. We showed that CD4^+^CD25^+^Foxp3^+^ T cells generated in the co-cultures with MSCs expressed significantly lower levels of helios and Nrp1 when compared to the Th1 or Th17 differentiated T cells cultured in absence of MSCs. These results strongly suggest that MSCs might generate iTreg cells rather than stimulating the expansion of nTreg cells (Figure 4B).

Finally, in a functional test, we assessed the suppressive capacity of conditioned Th1 or Th17 (Th1^cond^ or Th17^cond^) cells obtained after MSC addition at day 0 or 2 of the T cell differentiation processes (Figure 5B). By using different ratios of Th^cond^ cells:CD4^+^ (1:1; 1:2 or 1:5), we showed a dose dependent capacity of the conditioned cells to inhibit the proliferation of activated CD4^+^ T cells (Figure 5B). In contrast, conditioned T cells obtained from the co-cultures of mature Th1 or Th17 cells with MSCs did not modulate the proliferation of ConA activated T cells (data not shown).

### PGE2, TGF-β1 and IL-10 are up-regulated in the cocultures of MSCs with Th1- or Th-17 cells

**Figure 6:**
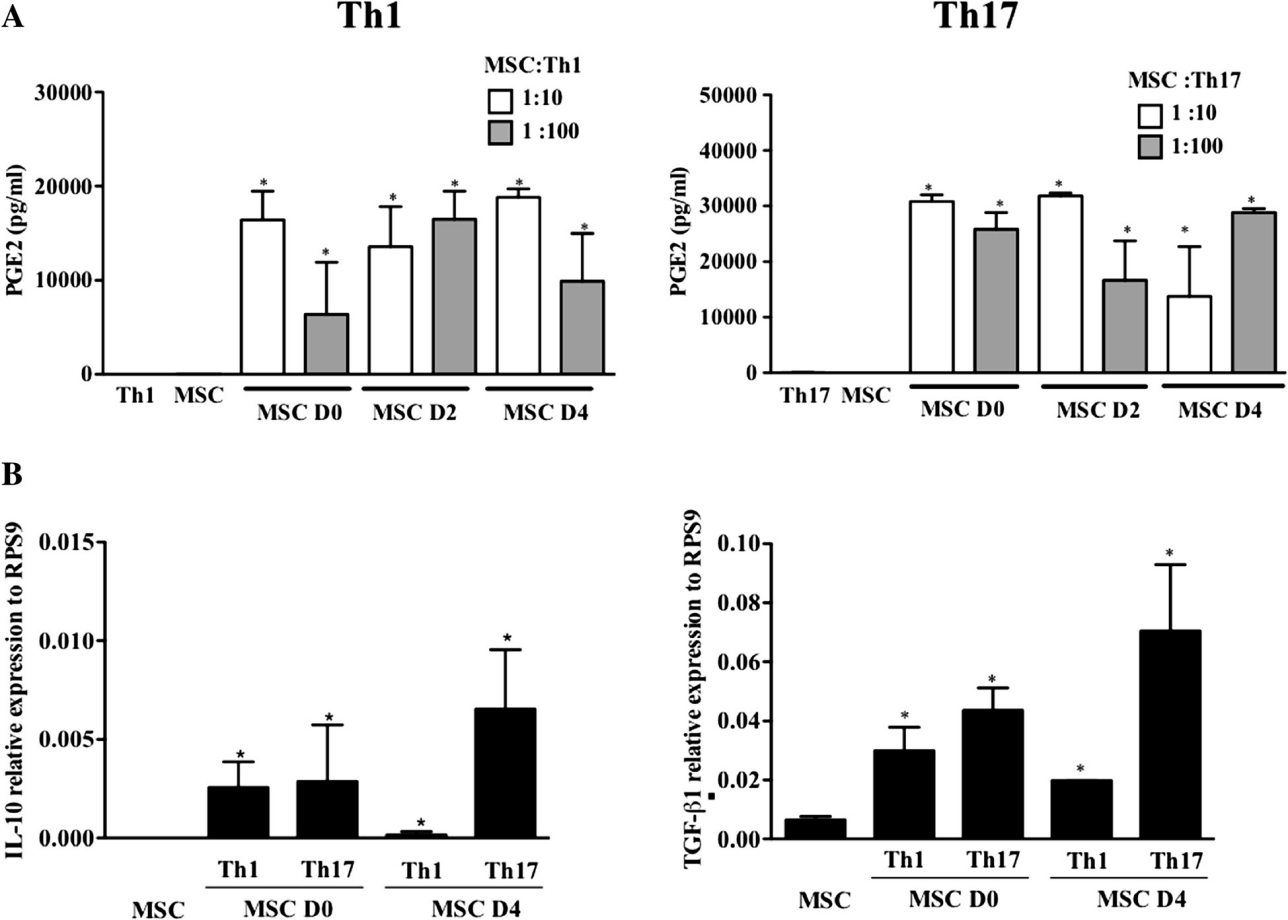
**PGE2, IL-10 and TGF-β1 expression on MSCs co-cultured with Th1 and Th17 cells. A)** PGE2 was quantified in the supernatants of MSCs co-cultured in the presence or absence of Th1 and Th17 cells. **B)** IL-10 and TGF-β1 expression was quantified in the supernatants of MSCs cultured in contact with Th1 and Th17 cells at different stages of the differentiation process (Day 0 and 4) ** = *P* <0.01, * = *P* <0.05, comparing to MSCs as the control group. All the values represent means ± SED of three independent experiments. MSCs, mesenchymal stem cells; PGE2, prostaglandin E2; TGF-β1, transforming growth factor β; Th, T helper.

Several studies have shown that soluble factors including PGE2, TGF-β1 and IL-10 are directly involved in Treg cell induction [27]. Therefore, we quantified PGE2 production in the supernatants of the co-cultures of MSCs with differentiating or mature Th1 or Th17 cells (Figure 6A). Compared to MSCs, Th1 or Th17 cells cultured alone, the secretion of PGE2 was significantly increased in the supernatants of the co-cultures. No significant difference was observed between the MSC:T cell ratios tested or between differentiating or mature Th1 or Th17 cells. Then, using quantitative PCR we measured the expression levels of these molecules in MSCs after co-culture with either differentiating or mature Th1 and Th17 cells. We found that all MSC co-cultures tested were able to increase the mRNA expression levels of TGF-β1 and IL-10 in MSCs (Figure 6B).

### MSCs inhibit EAE progression and increased the CD4^+^CD25^+^Foxp3^+^ Treg population

**Figure 7:**
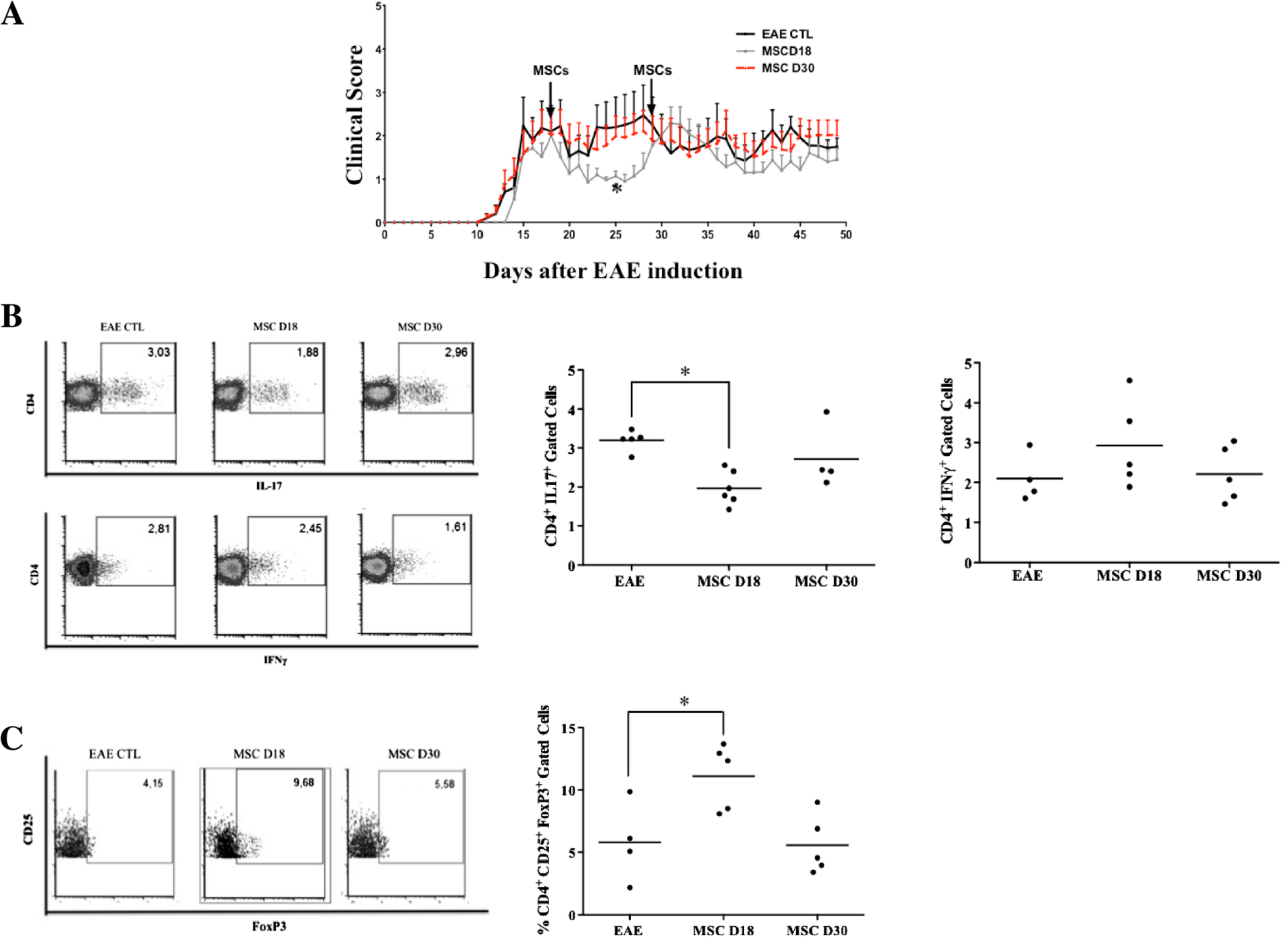
**Therapeutic effects and Th1, Th17 and CD4 + CD25 + Foxp3 + Treg populations in experimental autoimmune encephalomyelitis mice treated with MSCs. A)** Early intravenous injection of MSCs ameliorates EAE. MSCs were injected at days 18 and 30 after immunization as shown by arrows (1 × 10^6^ MSC/mice). **B)** CD4^+^IL17^+^ and CD4^+^IFN-γ^+^ cells in lymph nodes. MSCs significantly reduced the percentage of Th17 cells when injected at day 18 of the disease. **C)** CD4^+^CD25^+^Foxp3^+^ T cells in lymph nodes. MSCs induce CD4^+^CD25^+^Foxp3^+^ cells when injected at day 18 of the disease. Control: EAE mice without treatment. MSCD18: EAE mice treated with MSCs injected 18 days post-immunization. MSCD30: EAE mice treated with MSCs injected 30 days post-immunization. * = *P* <0.05, compared to EAE untreated mice as control group. All the values represent means ± SED of n = 5 control condition and n = 6 for MSCs treated mice. EAE, experimental autoimmune encephalomyelitis; MSCs, mesenchymal stem cells; Th, T helper; Treg, regulatory T cells.

Finally, we addressed whether MSCs could affect the generation of Th1, Th17 and CD4^+^CD25^+^Foxp3^+^ Treg cells in a T-cell-mediated autoimmune disease such as EAE. MSCs were administrated at day 18 and 30 after EAE induction in mice and the clinical scores were recorded daily until day 50. Compared to the control group, we observed an early improvement of clinical score when MSCs were administrated at day 18 (Figure 7A). This beneficial effect was observed shortly after MSC injection and maintained for ten days. However, MSC administration at day 30 did not improve the EAE symptoms. Then, the percentage of CD4^+^IFN-γ^+^, CD4^+^ IL-17^+^ and CD4^+^CD25^+^Foxp3^+^ T cells in lymph node cells and splenocytes were measured at day 50. We observed a significant decrease of Th17 cells and an increase of CD4^+^CD25^+^Foxp3^+^ T cells only in the lymph nodes of EAE mice that received MSCs at day 18 (Figure 7B and 7C). Compared to the control group, the percentage of Th1 cells was not modified (Figure 7B) and the different T cell sub-populations studied were not affected in the spleens after the MSC treatments (data not shown).

## Discussion

The capacity of MSCs to suppress the proliferation of T-cells has opened new perspectives for their clinical use. Currently, it is well established that MSCs rely on different mechanisms to exert their suppressive properties. One important mechanism is the capacity of MSCs to generate functional Treg cells, as shown in the last decade. The demonstration that direct MSC–T cell interactions induce T cells with regulatory properties has arisen from a number of studies describing the generation of Treg populations with typical phenotypic characteristics [10,11,28]. Later on, Prevosto *et al*. demonstrated that CD4^+^CD25^+^Foxp3^+^ cells, generated from PBMC after three days of culture with MSCs, were functional and inhibited the proliferation of alloantigen-activated T cells [29]. Another study established that MSCs were able to induce Treg cells from CD4^+^ T lymphocytes via cell contact as well as a non-redundant contribution of PGE2 and TGF-β1 [8]. These and other studies confirmed that Treg cell generation was contact- and soluble factors-dependent and priming of MSCs was required for an immunosuppressive effect [30]. However, the MSC potential to stimulate the proliferation of natural Tregs or to induce Tregs when co-cultured with Th1 or Th17 cell subsets has not been investigated. Therefore, we explored the ability of MSCs to induce functional CD4^+^CD25^+^Foxp3^+^ regulatory cells, when cultured in the presence of activated CD4^+^ T cells submitted to Th1 or Th17 skewing conditions. We first showed that MSCs strongly suppress the proliferation, activation and differentiation of Th1 and Th17 cells when added at early stages of the differentiation processes regardless of the cell ratio tested. However, MSCs added at the highest ratio in the presence of fully differentiated Th1 and Th17 cells were able to significantly reduce the percentage of the mature T cells as well as their proliferation rate. Moreover, we showed for the first time that under these culture conditions, MSCs were able to generate iTreg cells expressing low levels of Nrp 1 and helios. The generation of CD4^+^CD25^+^Foxp3^+^ regulatory T cells was not observed when MSCs were added to mature Th1 or Th17 cells. Furthermore, we showed that the latter iTreg cells were functional and suppressed the proliferation of activated CD4^+^ T cells.

It is now well established that the generation of Treg cells is mainly induced by the presence of TGF-β1 and IL-10 cytokines [31,32]. It has also been shown that PGE2 is involved in increased expression of Foxp3 in T-CD4^+^ cells [33] and that MSCs are able to produce PGE2, TGF-β1 and IL-10 [8,29]. Therefore, we determined the expression levels of IL-10 and TGF-β1 in MSCs and the concentration of IL-10 and PGE2 in the supernatants of MSCs co-cultured with Th1 or Th17 cells. In parallel to the Treg generation, we found that IL-10 production was significantly increased in supernatants of the co-culture when MSCs were added at day 0 or 2 of the differentiation processes. Along the same line, under the same conditions we reported a significant increase of IL-10 and TGF-β1 mRNA expression level in MSCs. However, we did not find an increase of IL-10 production in the supernatants of MSCs when cultured with fully differentiated Th1 or Th17 cells. We also showed an increase of PGE2 production in the supernatants of MSCs cultured with both differentiating or mature Th1 and Th17 cells. This observation supports other studies revealing that PGE2, TGF-β1 and IL-10 may play a critical role in the induction of Treg cells by MSCs [8,19].

Finally, we used an experimental murine model of EAE to evaluate the effect of MSCs injected at different time points post-immunization on the percentage of Th1, Th17 and CD4^+^CD25^+^Foxp3^+^ Treg cells. Since a single injection of MSCs at an early stage of the disease has been shown to improve EAE symptoms [34], in this study we performed one injection of MSCs at different stages of EAE. Our results demonstrated that MSCs were able to improve the clinical signs of EAE only when they were administrated at the peak of the disease. This therapeutic effect was associated with both a significant decrease of Th17 cell numbers and an increased percentage of CD4^+^CD25^+^Foxp3^+^ Treg cells in the lymph nodes. Interestingly, MSCs injected at the later time point neither diminished the percentage of Th17 cells nor increased the percentage CD4^+^CD25^+^Foxp3^+^ Treg cells. No effect was observed on Th1 cells.

Contradictory results have been previously reported following MSC injection showing either an increase of the Treg population associated with a therapeutic effect or describing an absence of any beneficial effect in different animal models of autoimmune diseases [14,35,36]. In the present study, we confirmed the generation of Treg cells following MSC injection in parallel with an improvement in the clinical signs of EAE.

## Conclusion

Taken together, these data demonstrate that MSCs are able to induce functional Treg cells during the differentiation process of Th1 and Th17 cells. The generation of Treg cells was associated with an increase of IL-10 production in the supernatant of the co-cultures and resulted in the inhibition of the proliferation, activation and differentiation of Th1 and Th17 cells. These data were confirmed in the EAE model, in which the therapeutic effect of MSCs on disease progression was associated with an increase of the percentage of Treg cells when MSCs were administrated at a specific time point during disease progression.

## Abbreviations

CFSE: Carboxyfluorescein succinimidyl ester; ConA: Concanavalin A; EAE: Experimental autoimmune encephalomyelitis; ELISA: Enzyme-linked immunosorbent assay; FACS: Fluorescence-activated cell sorting; FCS: Fetal calf serum; Fox: Forkhead box; IDO: Indoleamine 2,3-dioxygenase; IFN: Interferon; IL: Interleukin; iTreg: Induced Treg; MOG: Myelin oligodendrocyte glycoprotein; MSC: Mesenchymal stem cell; NO: Nitric oxide; Nrp1: Neuropilin 1; nTreg: Natural Treg; PBMC: Peripheral blood mononuclear cells; PBS: Phosphate-buffered saline; PCR: Polymerase chain reaction; PGE2: Prostaglandin E2; PMA: Phorbolmyristate acetate; Treg: Regulatory T cells; TGF-β1: Transforming growth factor β1; Th: T helper.

## Competing interests

The authors declare that they have no competing interests.

## Authors’ contributions

PL-C participated in the performance of all experiments, interpreted and analyzed the results and wrote the manuscript; MK and JB-A performed the EAE experiments and participated in data analysis; RC participated in Th1 differentiation experiments and helped in data analysis; EN-L participated in Th17 differentiation experiments and helped in data analysis; GT performed PCR experiments for Treg characterization and participated in data analysis; DN participated in the conception and design of the experiments and critically revised the content of the manuscript; CJ participated in the conception and design of the experiments; FF participated in the design of the experiments and critically revised the content of the manuscript; FD and FC designed the research, interpreted the data and wrote the manuscript. All authors read and approved the final manuscript.
